# Supplementary material for: Impact of Antibiotic Authorisation at Three Provincial Hospitals in Thailand: Results from a Quasi-Experimental Study
Source: Antibiotics (Basel). 2022 Mar 7;11(3):354. doi: 10.3390/antibiotics11030354 (PMC8944558; doi:10.3390/antibiotics11030354)
Supplement: Supplementary file 1 [file antibiotics-11-00354-s001.zip › antibiotics-1600530-supplementary.pdf]

Supplementary data

Impact of Antibiotic Authorisation at Three Provincial Hospitals in Thailand: Results from a Quasi-experimental Study

Walaiporn Wangchinda [1], Jintana Srisompong [2], Sunee Chayangsu [3], Darat Ruangkriengsin [4], Visanu Thamlikitkul [1], Pornpan Koomanachai [1], Rujipas Sirijatuphat [1] and Pinyo Rattanaumpawan [1,*]

[1] Division of Infectious Diseases and Tropical Medicine, Department of Medicine, Faculty of Medicine Siriraj Hospital, Mahidol University, Bangkok 10700, Thailand; walaiporn.wan@mahidol.ac.th (W.W.); visanu.tha@mahidol.ac.th (V.T.); pornpan.koo@mahidol.ac.th (P.K.); rujipas.sir@mahidol.ac.th (R.S.)
[2] Internal Medicine Unit, Surat Thani Hospital, Surat Thani 84000, Thailand; jint9839@gmail.com
[3] Internal Medicine Unit, Surin hospital, Surin 32000, Thailand; chayangsu.sunee@gmail.com
[4] Internal Medicine Unit, Sa Kaeo Crown Prince Hospital, Sa Kaeo 27000, Thailand; darat_r@yahoo.com
* Correspondence: E-mail: pinyo.rat@mahidol.ac.th

Table S1. Details of days of antimicrobial therapy during the pre- and post-implementation periods.

Variables	Total)*n*=1,802(mean±SD	Pre)*n*=901(mean±SD	Post)*n*=901(mean±SD	*p*-Value
All antimicrobials	11.73 ± 10.99	12.22 ± 10.59	11.25 ± 11.36	0.06
Targeted antimicrobials	6.94 ± 5.55	7.64 ± 6.10	6.24 ± 4.84	<0.001
**Penicillins**	0.12 ± 1.15	0.14 ± 1.16	0.11 ± 1.13	0.62
• Penicillin	0.02 ± 0.58	0.01 ± 0.17	0.03 ± 0.81	0.42
• Ampicillin	0.07 ± 0.82	0.08 ± 0.93	0.06 ± 0.68	0.54
• Amoxicillin	0.004 ± 0.19	0	0.01 ± 0.27	0.32
• Cloxacillin	0.03 ± 0.52	0.05 ± 0.65	0.02 ± 0.33	0.16
**Cephalosporins**	1.52 ± 4.57	1.06 ± 3.80	1.99 ± 5.18	<0.001
• Cefazolin	0.04 ± 0.74	0.04 ± 0.69	0.05 ± 0.80	0.85
• Ceftriaxone	0.69 ± 2.87	0.50 ± 2.79	0.88 ± 2.93	0.004
• Cefixime	0.21 ± 2.45	0.15 ± 1.39	0.27 ± 3.18	0.32
• Cefdinir	0.05 ± 0.65	0.04 ± 0.65	0.05 ± 0.65	0.72
• Cefepime	0.54 ± 2.27	0.33 ± 1.68	0.74 ± 2.71	<0.001
**Carbapenems**	4.72 ± 5.77	5.11 ± 6.35	4.34 ± 5.10	0.005
• Ertapenem	0.57 ± 2.43	0.66 ± 2.83	0.47 ± 1.94	0.11
• Meropenem	4.02 ± 5.55	4.32 ± 6.05	3.72 ± 4.98	0.02
• Imipenem	0.14 ± 1.19	0.13 ± 1.27	0.15 ± 1.10	0.72
**Beta-lactam/beta-lactamase inhibitors**	2.83 ± 4.88	3.19 ± 5.24	2.47 ± 4.46	0.002
• Amoxicillin/clavulanic acid	0.37 ± 2.19	0.37 ± 2.18	0.36 ± 2.19	0.90
• Ampicillin/sulbactam	0.06 ± 0.89	0.07 ± 0.84	0.05 ± 0.93	0.73
• Piperacillin/tazobactam	2.22 ± 3.78	2.54 ± 4.17	1.90 ± 3.33	<0.001
• Cefoperazone/subactam	0.18 ± 1.62	0.21 ± 1.78	0.16 ± 1.43	0.47
**Aminoglycosides**	0.05 ± 0.69	0.07 ± 0.78	0.04 ± 0.59	0.41
• Gentamicin	0.01 ± 0.21	0.01 ± 0.30	0	0.18
• Amikacin	0.05 ± 0.66	0.05 ± 0.72	0.04 ± 0.59	0.67
**Fluoroquinolones**	0.49 ± 2.20	0.51 ± 2.29	0.48 ± 2.11	0.79
• Norfloxacin	0.01 ± 0.20	0.01 ± 0.28	0	0.16
• Ciprofloxacin	0.29 ± 1.67	0.33 ± 1.87	0.24 ± 1.43	0.23
• Levofloxacin	0.20 ± 1.46	0.16 ± 1.29	0.24 ± 1.60	0.24
**Macrolides**	0.05 ± 1.02	0.08 ± 1.39	0.02 ± 0.38	0.21
• Clarithromycin	0.02 ± 0.95	0.05 ± 1.34	0	0.27
• Azithromycin	0.02 ± 0.37	0.03 ± 0.36	0.02 ± 0.38	0.52
**Other antimicrobial groups**	1.91 ± 6.57	2.06 ± 5.77	1.76 ± 7.28	0.34
• Colistin	0.76 ± 3.22	0.88 ± 3.10	0.64 ± 3.33	0.12
• Clindamycin	0.26 ± 1.91	0.28 ± 2.17	0.24 ± 1.61	0.69
• Cotrimoxazole	0.09 ± 1.29	0.09 ± 1.37	0.08 ± 1.20	0.88

• Doxycycline	0.02 ± 0.50	0.04 ± 0.64	0.01 ± 0.30	0.26
• Fosfomycin	0.30 ± 2.45	0.26 ± 1.71	0.34 ± 3.01	0.49
• Metronidazole	0.27 ± 2.88	0.21 ± 2.16	0.32 ± 3.44	0.38
